# Photo-Curing Kinetics of 3D-Printing Photo-Inks Based on Urethane-Acrylates

**DOI:** 10.3390/polym14152974

**Published:** 2022-07-22

**Authors:** Hadi Bakhshi, Guanxing Kuang, Franziska Wieland, Wolfdietrich Meyer

**Affiliations:** 1 Department of Life Science and Bioprocesses, Fraunhofer Institute for Applied Polymer Research IAP, Geiselbergstraße 69, 14476 Potsdam, Germany; wolfdietrich.meyer@iap.fraunhofer.de; 2 Department of Functional Polymer Systems, Fraunhofer Institute for Applied Polymer Research IAP, Geiselbergstraße 69, 14476 Potsdam, Germany; guanxingk@hotmail.com (G.K.); franziska.wieland@iap.fraunhofer.de (F.W.)

**Keywords:** 3D-printing photo-inks, urethane-acrylates, photopolymerization kinetics, photo-DSC, thermomechanical properties

## Abstract

In this study, photo-curing kinetics for urethane-acrylate-based photo-inks for 3D printing were evaluated using a photo-differential scanning calorimetry analysis. Initially, the photopolymerization kinetics of di- and monofunctional monomers were separately studied at different temperatures (5–85 °C). Later, the photo-curing kinetics and mechanical properties of photo-inks based on different monomer mixtures (40/60–20/80) were evaluated. The results showed that urethane-dimethacrylate (UrDMA) and urethane-acrylate (UrA) had no light absorption in the region of 280–700 nm, making them a proper crosslinker and a reactive diluent, respectively, for the formulation of 3D-printing photo-inks. The kinetics investigations showed a temperature dependency for the photo-curing of UrDMA, where a higher photopolymerization rate (*R_p,max_*: from 5.25 × 10^−2^ to 8.42 × 10^−2^ 1/s) and double-bound conversion (*DBC_total_*: from 63.8% to 92.2%) were observed at elevated temperatures (5–85 °C), while the photo-curing of UrA was independent of the temperature (25–85 °C). Enhancing the UrA content from 60% to 80% in the UrDMA/UrA mixtures initially increased and later decreased the photopolymerization rate and conversion, where the mixtures of 30/70 and 25/75 presented the highest values. Meanwhile, increasing the UrA content led to lower glass transition temperatures (*T_g_*) and mechanical strength for the photo-cured samples, where the mixture of 30/70 presented the highest maximum elongation (*ε_max_*: 73%).

## 1. Introduction

Photo-curable inks (photo-inks) play a key role in light-based, 3D-printing technologies, e.g., stereolithography (SLA), digital light processing (DLP), liquid crystal precision (LCP), and digital light synthesis (DLS) or continuous liquid interface production (CLIP) [1]. Under irradiation, liquid photo-ink consisting of mono- and multifunctional monomers and a photoinitiator undergoes a photopolymerization reaction and transforms into a solid polymeric network within a very short time. Recently, urethane-acrylates have been used to formulate 3D-printing photo-inks [2,3,4,5,6,7,8,9,10]. The intermolecular hydrogen bonding between the urethane groups allows the pre-association of urethane-acrylate molecules and, consequently, 3–6 times faster photo-curing in comparison to their corresponding nonhydrogen-bonding acrylates [11,12]. Moreover, hydrogen bonding improves the mechanical properties of 3D-printed constructs [13,14].

The main research trend for light-based, 3D-printing technologies is focused on the development of advanced photo-ink formulations and innovative processes to obtain high-performance constructs for cutting-edge applications [1,15]. Meanwhile, the fundamental investigation of the photopolymerization kinetics of photo-inks has been less reported [10,16,17,18]. To better understand the characteristics of photo-inks and, consequently, to improve the quality and performance of 3D-printed constructs, studying photo-curing kinetics is essential. Recently, Jiang et al. [17] reported that higher UV intensity improved the photo-curing rate (*R_p_*) and the double-bound conversion (DBC) of a photo-ink based on 1,6-hexamethylene diacrylate, while longer exposure time partially enhanced the DBC. Deng et al. [10] reported that enhancing the concentration of the photoinitiator improved the *R_p_* and DBC values for photo-ink formulations based on urethane-acrylates. According to Kim et al. [18], higher temperature led to higher *R_p_*, DBC, and glass transition temperature (*T_g_*) values for a commercial UV-curable, 3D-printing acrylic resin (VeroWhitePlus RGD 835, Stratasys Ltd., Minneapolis, MN, USA). Bachmann et al. [16] studied the photo-curing kinetics of three commercial photo-inks for DLS 3D printing under different wavelengths, light intensities, temperatures, relative humidities, and atmospheric oxygen concentrations. One of their observations was the improvement of *R_p_* by decreasing the atmospheric oxygen concentration, which is the reason behind a shorter printing time under an inert atmosphere.

In this work, initially, the photopolymerization kinetics of a difunctional and a monofunctional urethane-acrylate monomer, respectively, as a crosslinker and a reactive diluent in 3D-printing photo-ink formulations are studied separately at different temperatures (5–85 °C). For this purpose, the released heat during the exothermic photopolymerization reactions is measured through photo-differential scanning calorimetry (photo-DSC) analysis. Later, the photo-curing of photo-inks based on different mixtures of these monomers (40/60–20/80), as well as the thermomechanical properties of corresponding photo-cured samples, are investigated.

## 2. Experimental

### 2.1. Materials

7,7,9(or 7,9,9)-Trimethyl-4,13-dioxo-3,14-dioxa-5,12-diazahexadecane-1,16-diol dimethacrylate (UrDMA) as a crosslinker, 2-[[(butylamino)carbonyl]oxy]ethyl 2-propenoate (UrA) as a reactive diluent, and ethyl (2,4,6-trimethylbenzoyl)phenylphosphinate (TPOL) as a photoinitiator were received from German industrial suppliers and used without any treatment.

### 2.2. Instruments

UV–Vis spectroscopy of the monomers and photoinitiator in an acetonitrile solution (0.8 g/L) was performed using a PerkinElmer instrument (Lambda 950, Norwalk, CT, USA) operating in the range of 200–700 cm^−1^. Acetonitrile was used as a blank solution.

A DSC from Netzsch (DSC 204 F1 Phoenix, Mannheim, Germany) equipped with a UV-curing spot system (OmniCure® S2000, Lumen Dynamics, Toronto, ON, Canada) was employed to study the photo-curing of the samples. The experiments were conducted in an isothermal mode (5, 25, 45, 65, or 85 °C) with a UV intensity of 1 W/m^2^ under N_2_ atmosphere. The UV intensity was measured with a radiometer (R2000 Radiometer, Lumen Dynamics, Toronto, ON, Canada). The samples were weighted in uncovered aluminum pans in darkness and analyzed immediately. An empty, uncovered aluminum pan was employed as a reference. The irradiation step was prolonged up to 3 min for the full photo-curing of the samples and reached a plateau region, which was employed as a baseline for the peak integrations. The *T_g_* of the photo-cured samples was determined using DSC measurements in the temperature range of −50 to 200 °C with the heating rate of 10 °C/min under N_2_ atmosphere. The *T_g_* values were extracted from the middle point of the baseline change in the first heating cycle.

The tensile properties of the photo-cured specimens were measured with a Zwick/Roell machine (model Z020, Ulm, Germany) equipped with a load cell of 100 N at room temperature. Photo-cured samples with a thickness of 300–500 µm were cut into 8 × 2 cm^2^ pieces and analyzed with a clamping length of 34 mm and a crosshead speed of 17 mm/min. The reported values were the averages of at least three specimens for each sample.

### 2.3. Methods 

The *R_p_* (1/s) and DBC (%) values as a function of time were calculated from the photo-DSC data using Equations (1)–(3) [7]:

(1) $$\Delta H_{theor}=\frac{f\cdot n\cdot \Delta H_{0}}{M_{w}}$$

(2) $$R_{p}=\frac{dH_{p}}{dt}/\Delta H_{theor}$$

(3) $$DBC=\frac{\Delta H_{p}}{\Delta H_{theor}}\times 100$$

where *ΔH_theor_* (J/g) is the theoretical total heat released during the complete polymerization of the monomers within the sample, *f* is the mass fraction of each monomer within the sample, *n* is the number of double bonds in each monomer, *ΔH_0_* (J/mol) is the standard heat of polymerization for either methacrylate (54.8 kJ/mol [19,20]) or acrylate (86.2 kJ/mol [20,21]), *M_w_* (g/mol) is the molecular weight of each monomer, *dH_p_/dt* (J/s·g or W/g) is the normalized heat flow per second, and *ΔH_p_* (J/g) is the heat generated from the start of photo-curing up to a certain time that is obtained from the integration of the thermograms.

To prepare tensile test specimens, formulations based on UrDMA/UrA mixtures (40/60–20/80) containing TPOL (1 wt.%) were cast with a thickness of 700 µm on a PET substrate using an adjustable applicator and cured under a UV lamp (145 mW/m^2^) for 60 s. After washing in isopropanol, the backs of the films were post-cured for 60 s.

## 3. Results and Discussion

**Scheme 1:**
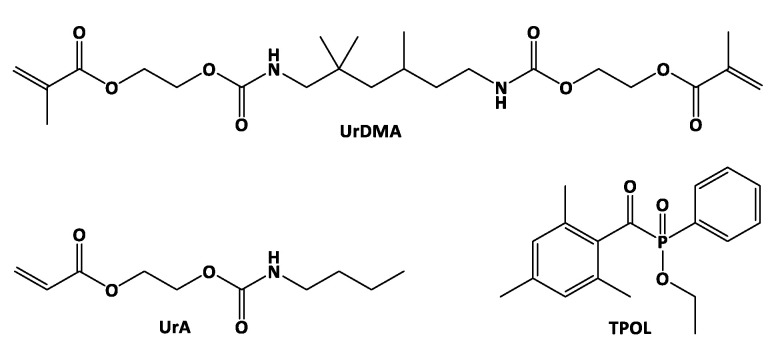
The chemical structures of UrDMA, UrA, and TPOL.

The chemical structures of the used chemicals are depicted in Scheme 1. UrDMA as a viscous, difunctional monomer (*η* = 9500 mPa·s) was used in the photo-ink formulations to improve the dimensional stability of the 3D constructs by generating a thermoset network structure [22,23]. Monofunctional UrA (*η* = 35 mPa·s) was employed as a reactive diluent to decrease the viscosity of the formulations, while resulting in flexibility for the 3D constructs [9,24]. TPOL as a photoinitiator was added to the formulations at 1 wt.% regarding the total weight of the monomers. TPOL has previously shown excellent biocompatibility, color stability, and 3D-printing accuracy compared to common photoinitiators, i.e., bis(2,4,6-trimethylbenzoyl)phenylphosphine oxide (BAPO) and (2,4,6-Trimethylbenzoyl)diphenylphosphine oxide (TPO) [25,26,27].

### 3.1. UV–vis Absorption

**Figure 1:**
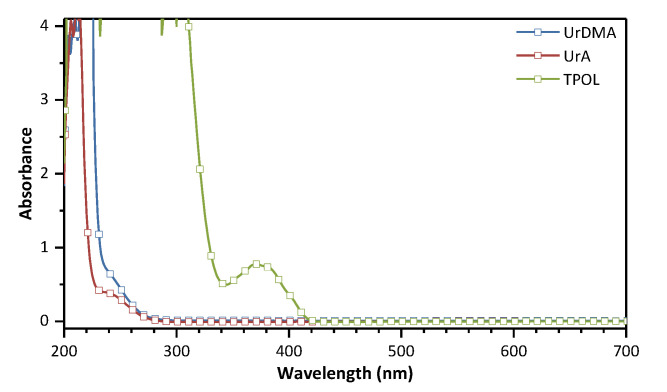
UV–vis spectra for UrDMA, UrA, and TPOL in acetonitrile solution (0.8 g/L).

Under a light, photoinitiator molecules absorb energy, convert to an excited state, and generate free radicals to initiate the photopolymerization of monomers. Before studying the photopolymerization kinetics of monomers, their UV–vis spectra in an acetonitrile solution were recorded (Figure 1). Both monomers (UrDMA and UrA) were transparent in the region of 280–700 nm, which is ideal for the photo-curing process since the whole irradiated light is accessible to the photoinitiator molecules. Meanwhile, TPOL showed absorption in the range of 200–420 nm, with two maximums at 273 and 371 nm. The absorption at 371 nm makes TPOL active for commercial DLP 3D printers and post-curing units, which mainly function at 365, 385, or 405 nm.

### 3.2. Photo-Curing

The most common type of photopolymerization for 3D-printing applications is through the free-radical mechanism, which involves three main steps: (1) initiation, (2) propagation, and (3) termination [15]. In the initiation step, the photoinitiator molecules are disintegrated under light irradiation and produce reactive radical fragments. In the propagation step, these radical species react with the double bonds (C=C) of monomer molecules to generate growing polymer chains. The growth of these polymer chains continues simultaneously with the addition of further monomers and the formation of macroradicals. In the termination step, the growing polymer chains are deactivated through combination, radical disproportionation, or chain transfer reactions [15].

**Figure 2:**
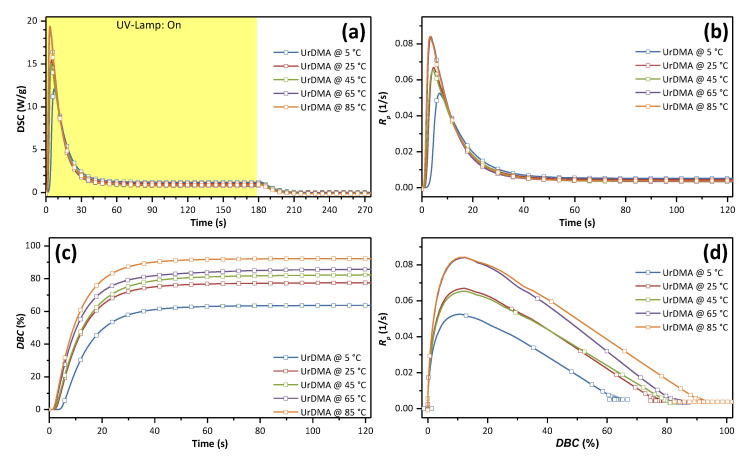
Photo-curing rates and conversions for UrDMA containing TPOL (1 wt.%) under N_2_ atmosphere at different temperatures obtained from photo-DSC data. The intensity of the UV lamp was 1 W/m^2^. (**a**) DSC vs. time; (**b**) *R_p_* vs. time; (**c**) *DBC* vs. time; (**d**) *R_p_* vs. *DBC*.

**Figure 3:**
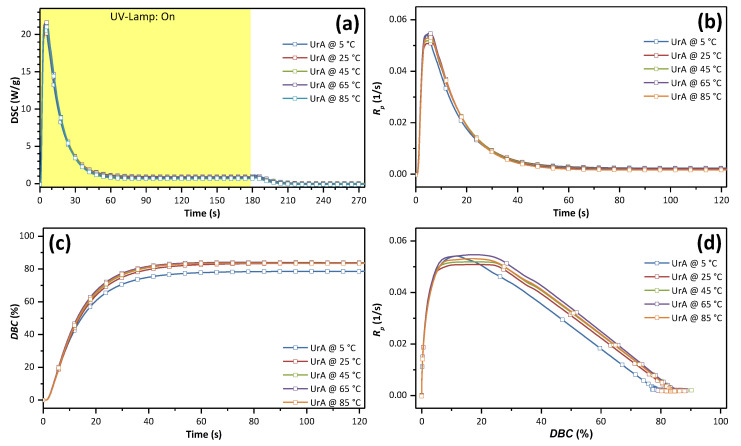
Photo-curing rates and conversions for UrA containing TPOL (1 wt.%) under N_2_ atmosphere at different temperatures obtained from photo-DSC data. The intensity of the UV lamp was 1 W/m^2^. (**a**) DSC vs. time; (**b**) *R_p_* vs. time; (**c**) *DBC* vs. time; (**d**) *R_p_* vs. *DBC*.

Photo-DSC analyses have been widely utilized to evaluate the photo-activity of 3D-printing photo-inks [7,16,17]. Primarily, the photopolymerization kinetics of difunctional UrDMA and monofunctional UrA were studied separately in the isothermal mode at different temperatures, i.e., at 5, 25, 45, 65, or 85 °C (Figure 2a and Figure 3a). The *R_p_* and DBC values as a function of irradiation time were calculated from the photo-DSC data according to Equations (1)–(3) (Figure 2b–d and Figure 3b–d). For both UrDMA and UrA, the *R_p_* value, an overall value of the propagation and termination rates, initially increased and later decreased during the photo-curing process (Figure 2b and Figure 3b), which are known as the auto-acceleration and auto-deceleration phenomena, respectively. This observation was due to the gradual increase in viscosity with conversion and, consequently, the decrease in molecular mobility of the monomers and macroradicals within the photo-curing mixture over time [28,29]. Initially, at a low viscosity, the *R_p_* of the liquid monomers was constant and dependent on the double-bond activity (chemistry-controlled). Later, at medium viscosity, the coupling of macroradicals in the photo-curing mixture for termination was diffusion-limited when the monomers were still mobile for propagation, and thus, the *R_p_* increased (auto-acceleration). Finally, at a high viscosity, when the photo-curing mixture was transforming into a rubbery, glassy network (gel point), the diffusion of monomers to reach the macroradicals was significantly restricted, and therefore, the *R_p_* decreased (auto-deceleration) [28,29]. For both UrDMA and UrA, the DBC sharply increased over the first 20–30 s of the photo-curing process and remained constant after 40 s (Figure 2c and Figure 3c). The total double-bound conversion (*DBC_total_*) values obtained from the total generated photo-curing heat (*ΔH_p,total_*) were less than unity due to the gelation or vitrification phenomenon [28,29], which meant that all the double bonds did not react in the course of the photo-curing process (Table 1 and Table 2).

For difunctional UrDMA, the maximum photo-curing rate (*R_p,max_*) gradually increased from 5.25 × 10^−2^ to 8.42 × 10^−2^ 1/s by raising the photo-curing temperature (*T_p_*) from 5 to 85 °C (Table 1). Meanwhile, the gel-point time (*t_GP_*), the time to reach *R_p,max_*, decreased from 7.0 to 3.3 s, and the gel-point conversion (*DBC_GP_*), the conversion at *R_p,max_*, increased from 10.9% to 12.3%. These observations showed higher photo-activity leading to faster photopolymerization for UrDMA at elevated temperatures, which is in agreement with previous reports on multifunctional monomers [28,30,31,32]. The increase in *R_p,max_* values by raising the temperature can also be explained according to Arrhenius’ law (Equation (4)) [31]:

(4) $$k=k_{p}=A\times e^{-E_{a}/RT}$$

where *k* is the overall photopolymerization rate constant equal to the propagation rate constant (*k_p_*) at low conversions, *E_a_* is the activation energy, *A* is the frequency factor, *R* is the ideal gas constant, and *T* is the absolute temperature. According to Arrhenius’ law, a rise in temperature results in an increase in *k* and, consequently, *R_p,max_*.

On the other hand, the *DBC_total_* of UrDMA was improved from 63.8% to 92.2% (Table 1) due to lower viscosity and higher molecular mobility within the photo-curing mixture at higher temperatures, which is in agreement with previous reports [28,30,31,32]. Meanwhile, the times to reach 50%, 90%, and 95% of *DBC_total_* (*t*_50%_, *t*_90%_, and *t*_95%_, respectively) were shortened, which meant faster photo-curing processes at elevated temperatures. The *T_g_* of the photo-cured samples obtained from DSC measurements was improved from 63 to 122 °C, which was expected according to the *DBC_total_* trend, where more conversion for difunctional UrDMA resulted in networks with higher crosslinking densities, lower polymer chain flexibility, and consequently, higher *T_g_* values. It is worth mentioning that, for all the photo-cured samples, the *T_g_* values were higher than the *T_p_*, which meant that the conversion was restricted due to the transformation of the liquid monomer to a glassy network with insufficient molecular mobility [31].

For monofunctional UrA, the *R_p,max_* remained constant in the range of 5.08 × 10^−2^–5.47 × 10^−2^ 1/s by increasing the *T_p_* from 5 to 85 °C (Table 2). In the *T_p_* range of 25–85 °C, the *t_GP_*, *DBC_GP_,* and *DBC_total_* remained constant in the ranges of 5.2–5.7 s, 16.5–18.5%, and 83.3–83.8%, respectively. These phenomena meant that raising the temperature did not affect the photo-activity and conversion of UrA, which is in contrast with previous results about 2-hydroxyethyl acrylate (HEA) [11], undecyl amide *N*-ethyl acrylate [12], and 2-carboxyethyl acrylate [33], where the *R_p,max_* has decreased with increasing *T_p_* from 25 °C to 90 °C as a result of the dissociation of the intermolecular hydrogen bonding and disappearance of the pre-association of monomers. At 5°C, UrA displayed a shorter *t_GP_* (4.4 s) and lower *DBC_GP_* (11.9%) and *DBC_total_* (78.7%) values, which was attributed to the crystallization of UrA molecules with a melting point (*T_m_*) of 5 °C, leading to lower molecular mobility within the photo-curing mixture. This observation is in agreement with a previous report on crystalizable monomers [7].

In the *T_p_* range of 25–85 °C, *t*_50%_, *t*_90%_, and *t*_95%_ were shortened by increasing the temperature, which meant faster photo-curing processes at higher temperatures. The *T_g_* of the photo-cured samples remained constant at −11 °C in the *T_p_* range of 5–65 °C and then partially increased to −8 °C at a *T_p_* of 85 °C, which was expected due to almost the same *DBC_total_* values. For all the photo-cured samples, the *T_g_* values were lower than the *T_p_*, meaning the photo-curing mixture transformed from a low-viscosity liquid monomer into a rubbery network with flexible polymer chains. It is worth mentioning that, although the photo-cured monofunctional UrA was supposed to be a thermoplastic, the DSC analyses up to 200 °C did not show any exothermic melting peak.

**Figure 4:**
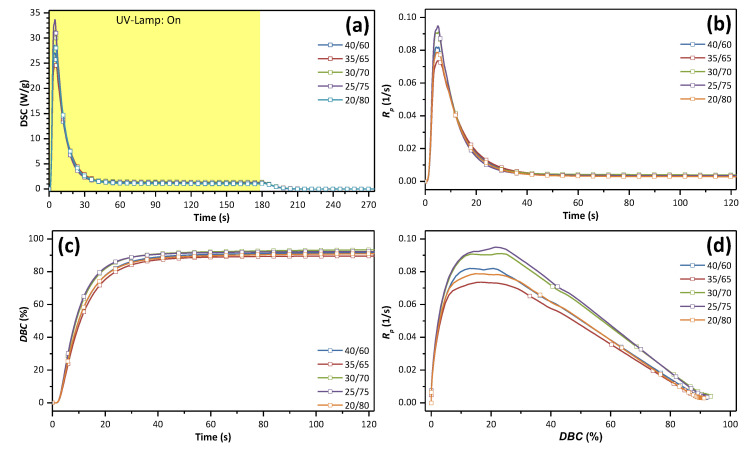
Photo-curing rates and conversions for UrDMA/UrA mixtures containing TPOL (1 wt.%) under N_2_ atmosphere at 25 °C obtained from photo-DSC data. The intensity of the UV lamp was 1 W/m^2^. (**a**) DSC vs. time; (**b**) *R_p_* vs. time; (**c**) *DBC* vs. time; (**d**) *R_p_* vs. *DBC*.

Later, the photopolymerization kinetics of different UrDMA/UrA mixtures (40/60–20/80) as bases for photo-inks were evaluated in isothermal mode at 25 °C (Figure 4 and Table 3). The *R_p,max_* and *DBC_total_* for the UrDMA/UrA mixtures were higher (7.35 × 10^−2^–9.50 × 10^−2^ 1/s and 90–92%, respectively) compared with pure UrDMA and UrA at 25 °C, while *t*_50%_, *t*_90%_, and *t*_95%_ were shorter. These observations showed higher photo-activity, leading to faster photopolymerization and higher conversion for the UrDMA/UrA mixtures.

By enhancing the content of the monofunctional reactive diluent (UrA) from 60% to 80% in the UrDMA/UrA mixtures, the *R_p,max_*, *t_GP_*, *DBC_GP_*, *DBC_total_* initially increased and then decreased, where the mixtures of 30/70 and 25/75 presented the highest values (Table 3). Meanwhile, *t*_50%_, *t*_90%_, and *t*_95%_ initially decreased and then increased. Since UrA has a lower viscosity (*η* = 35 mPa·s) than UrDMA (*η* = 9500 mPa·s), increasing the UrA content led to photo-curing mixtures with lower viscosity. At the same time, it resulted in lower average double-bound functionality within the photo-curing mixture and, consequently, later gelation. Both phenomena enhanced the molecular mobility within the photo-curing mixture, which explained the initial increasing trend for the *R_p,max_*, *t_GP_*, *DBC_GP_*, *DBC_total_* values, as well as the decreasing trend for the *t*_50%_, *t*_90%_, and *t*_95%_ values. Therefore, faster photopolymerization and higher conversion were observed for the mixtures of 30/70 and 25/75. The later decreasing trend for the *R_p,max_*, *t_GP_*, *DBC_GP_*, *DBC_total_* values and the increasing trend for the *t*_50%_, *t*_90%_, and *t*_95%_ values, especially for the mixture of 20/80, could be attributed to the higher deactivation of macroradicals through the combination of macroradicals and, thus, the limited auto-acceleration at a very low viscosity.

The *T_g_* of the photo-cured samples decreased from 23 to −11 °C by increasing the UrA content from 60% to 80%, which was expected due to the reduction in the average double-bound functionality, the crosslinking density of the polymeric network, and thus, the polymer chain flexibility. For all the photo-cured samples, the *T_g_* values were lower than 25 °C, meaning the photo-curing mixture transformed from a viscous liquid monomer into a rubbery network with flexible polymer chains.

### 3.3. Mechanical Properties

**Figure 5:**
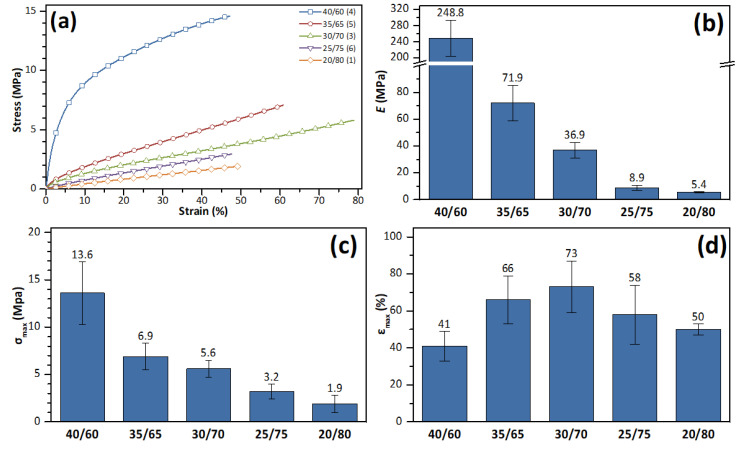
Stress–strain curves (**a**), tensile modulus (*E*, (**b**)), maximum tensile strength (*σ_max_*, (**c**)), and maximum elongation (*ε_max_*, (**d**)) of the photo-cured samples based on UrDMA/UrA mixtures containing TPOL (1 wt.%).

Enhancing the content of the monofunctional reactive diluent in photo-inks, which decreased the average double-bound functionality, could influence the mechanical properties of 3D-printed objects. The mechanical properties of the photo-cured samples were investigated with tensile tests (Figure 5). The results presented the typical stress–strain curves of a thermoset polymer (Figure 5a). Enhancing the UrA content from 60% to 80% led to a decrease in the tensile module (*E*) from 248.8 to 5.4 MPa (Figure 5b) and tensile strength (*σ_max_*) from 13.6 to 1.9 MPa (Figure 5c), which was expected due to the reduction in the crosslinking density of the polymeric network as a result of the fall in the average double-bound functionality within corresponding photo-curing mixtures. Meanwhile, the maximum elongation (*ε_max_*) initially increased from 41% to 73% (for the mixture of 30/70) and then decreased to 50% (Figure 5d). These observations are in agreement with a previous report about photo-cured elastomers based on mono- and difunctional urethane-acrylates [10].

## 4. Conclusions

UrDMA and UrA with no light absorption in the region of 280–700 nm were presented as a proper crosslinker and a reactive diluent, respectively, for the formulation of 3D-printing photo-inks. Kinetics investigations showed a temperature dependency for the photo-curing of UrDMA, where higher photopolymerization rates, conversion, and *T_g_* were observed at elevated temperatures, while the photo-curing of UrA was independent of temperature (25–85 °C). Enhancing the UrA content from 60% to 80% in the UrDMA/UrA mixtures initially increased and later decreased the photopolymerization rate and conversion, where the mixtures of 30/70 and 25/75 presented the highest values. Meanwhile, increasing the UrA content led to lower *T_g_*, *E*, and *σ_max_* values for the photo-cured samples, where the mixture of 30/70 presented the highest *ε_max_* value. For future investigations, studying the effects of other parameters, such as photoinitiator type and concentration, not only on photo-curing kinetics but also on the 3D printability of photo-inks may be interesting. Employing other instruments, such as online FTIR or a rheometer, may be helpful to better understand the chemical changes during the photo-curing process.

## Figures and Tables

**Table 1:** Photo reactivity of UrDMA containing 1 wt.% TPOL under N_2_ atmosphere at different temperatures obtained from photo-DSC data.

*T_p_* (°C)	*R_p,max_* (1/s)	*t_GP_* (s)	*DBC_GP_* (%)	*ΔH_p,total_* (J/g)	*DBC_total_* (%)	*t*_50%_ (s)	*t*_90%_ (s)	*t*_95%_ (s)	*T_g_* (°C)
5	5.25 × 10^−2^	7.0	10.9	148.6	63.8	12.4	29.2	37.8	63
25	6.70 × 10^−2^	4.7	12.1	180.8	77.6	9.9	26.3	34.5	77
45	6.52 × 10^−2^	4.5	12.7	192.0	82.4	10.2	28.5	38.2	101
65	8.40 × 10^−2^	3.6	12.3	199.9	85.8	8.8	26.2	38.4	106
85	8.42 × 10^−2^	3.3	12.3	214.8	92.2	8.5	23.7	30.9	122

**Table 2:** Photo reactivity of UrA containing 1 wt.% TPOL under N_2_ atmosphere at different temperatures obtained from photo-DSC data.

*T_p_* (°C)	*R_p,max_* (1/s)	*t_GP_* (s)	*DBC_GP_* (%)	*ΔH_p,total_* (J/g)	*DBC_total_* (%)	*t*_50%_ (s)	*t*_90%_ (s)	*t*_95%_ (s)	*T_g_* (°C)
5	5.42 × 10^−2^	4.4	11.9	315.2	78.7	10.0	30.4	39.2	−11
25	5.08 × 10^−2^	5.7	17.8	334.8	83.6	11.2	30.4	39.3	−11
45	5.19 × 10^−2^	5.7	18.5	335.7	83.8	11.1	28.8	36.3	−11
65	5.47 × 10^−2^	5.2	17.1	335.2	83.7	10.6	27.3	33.9	−11
85	5.31 × 10^−2^	5.3	16.5	335.5	83.3	10.9	27.7	34.3	−8

**Table 3:** Photo reactivity of UrDMA/UrA mixtures containing 1 wt.% TPOL under N_2_ atmosphere at 25 °C obtained from photo-DSC data.

UrDMA/UrA	*R_p,max_* (1/s)	*t_GP_* (s)	*DBC_GP_* *(%)*	*ΔH_p,total_* (J/g)	*DBC_total_* (%)	*t*_50%_ (s)	*t*_90%_ (s)	*t*_95%_ (s)	*T_g_* (°C)
40/60	8.20 × 10^−2^	4.2	12.9	303.1	91.8	9.3	24.6	32.8	23
35/65	7.35 × 10^−2^	5.0	16.7	302.9	89.5	9.6	24.8	32.1	17
30/70	9.10 × 10^−2^	5.2	23.5	324.3	93.5	8.4	22.2	30.4	9
25/75	9.50 × 10^−2^	4.9	21.3	327.5	92.3	8.1	20.5	27.1	9
20/80	7.87 × 10^−2^	4.6	15.6	331.4	91.2	9.0	24.4	33.3	4

## Data Availability

Not applicable.

## References

[1] Quan H., Zhang T., Xu H., Luo S., Nie J., Zhu X. (2020). Photo-curing 3D printing technique and its challenges. Bioact. Mater..

[2] Tzeng J.-J., Yang T.-S., Lee W.-F., Chen H., Chang H.-M. (2021). Mechanical Properties and Biocompatibility of Urethane Acrylate-Based 3D-Printed Denture Base Resin. Polymers.

[3] Buchheit H., Bruchmann B., Stoll K., Mülhaupt R. (2021). Functionalized acrylic polyhydroxy urethanes as molecular tool box for photocurable thermosets and 3D printing. J. Appl. Polym. Sci..

[4] Peng S., Li Y., Wu L., Zhong J., Weng Z., Zheng L., Yang Z., Miao J.-T. (2020). 3D Printing Mechanically Robust and Transparent Polyurethane Elastomers for Stretchable Electronic Sensors. ACS Appl. Mater. Interfaces.

[5] Chen H., Lee S.-Y., Lin Y.-M. (2020). Synthesis and Formulation of PCL-Based Urethane Acrylates for DLP 3D Printers. Polymers.

[6] Kim S., Lee J., Han H. (2020). Synthesis of UV Curable, Highly Stretchable, Transparent Poly(urethane-acrylate) Elastomer and Applications Toward Next Generation Technology. Macromol. Res..

[7] Singh N., Bakhshi H., Meyer W. (2020). Developing non-isocyanate urethane-methacrylate photo-monomers for 3D printing application. RSC Adv..

[8] Baker M., Wang R., Damanik F., Kuhnt T., Ippel H., Dijkstra P., ten Cate T., Dias A., Moroni L. (2020). Biodegradable Poly(Ester) Urethane Acrylate Resins for Digital Light Processing: From Polymer Synthesis to 3D Printed Tissue Engineering Constructs. ChemRxiv.

[9] Soreni-Harari M., Pierre R.S., McCue C., Moreno K., Bergbreiter S. (2020). Multimaterial 3D Printing for Microrobotic Mechanisms. Soft Robot..

[10] Deng Y., Li J., He Z., Hong J., Bao J. (2020). Urethane acrylate-based photosensitive resin for three-dimensional printing of stereolithographic elastomer. J. Appl. Polym. Sci..

[11] Lee T.Y., Roper T.M., Jönsson E.S., Guymon C.A., Hoyle C.E. (2004). Influence of Hydrogen Bonding on Photopolymerization Rate of Hydroxyalkyl Acrylates. Macromolecules.

[12] Jansen J.F.G.A., Dias A.A., Dorschu M., Coussens B. (2003). Fast Monomers: Factors Affecting the Inherent Reactivity of Acrylate Monomers in Photoinitiated Acrylate Polymerization. Macromolecules.

[13] Barszczewska-Rybarek I.M. (2009). Structure–property relationships in dimethacrylate networks based on Bis-GMA, UDMA and TEGDMA. Dent. Mater..

[14] Lemon M.T., Jones M.S., Stansbury J.W. (2007). Hydrogen bonding interactions in methacrylate monomers and polymers. J. Biomed. Mater. Res. Part A.

[15] Bagheri A., Jin J. (2019). Photopolymerization in 3D Printing. ACS Appl. Polym. Mater..

[16] Bachmann J., Schmölzer S., Ruderer M.A., Fruhmann G., Hinrichsen O. (2021). Photo-differential scanning calorimetry parameter study of photopolymers used in digital light synthesis. SPE Polym..

[17] Jiang F., Drummer D. (2020). Curing Kinetic Analysis of Acrylate Photopolymer for Additive Manufacturing by Photo-DSC. Polymers.

[18] Kim Y.C., Hong S., Sun H., Kim M.G., Choi K., Cho J., Choi H.R., Koo J.C., Moon H., Byun D. (2017). UV-curing kinetics and performance development of in situ curable 3D printing materials. Eur. Polym. J..

[19] Assumption H.J., Mathias L.J. (2003). Photopolymerization of urethane dimethacrylates synthesized via a non-isocyanate route. Polymer.

[20] Anseth K.S., Wang C.M., Bowman C.N. (1994). Kinetic evidence of reaction diffusion during the polymerization of multi(meth)acrylate monomers. Macromolecules.

[21] Harikrishna R., Ponrathnam S., Rajan C.R., Tambe S.S. (2012). Photopolymerization of bis-aromatic and alicyclic based solid urethane acrylate macromonomer in the presence of large excess of reactive diluent. J. Therm. Anal..

[22] Xu Y., Wang H., Xie D. (2017). Preparation of new low viscosity urethane dimethacrylates for dental composites. J. Biomater. Sci. Polym. Ed..

[23] Barszczewska-Rybarek I. (2017). The role of molecular structure on impact resistance and bending strength of photocured urethane-dimethacrylate polymer networks. Polym. Bull..

[24] Weigand J.J., Miller C.I., Janisse A.P., McNair O.D., Kim K., Wiggins J.S. (2020). 3D printing of dual-cure benzoxazine networks. Polymer.

[25] Kim G.-T., Go H.-B., Yu J.-H., Yang S.-Y., Kim K.-M., Choi S.-H., Kwon J.-S. (2022). Cytotoxicity, Colour Stability and Dimensional Accuracy of 3D Printing Resin with Three Different Photoinitiators. Polymers.

[26] Zeng B., Cai Z., Lalevée J., Yang Q., Lai H., Xiao P., Liu J., Xing F. (2021). Cytotoxic and cytocompatible comparison among seven photoinitiators-triggered polymers in different tissue cells. Toxicol. Vitr..

[27] Steyrer B., Neubauer P., Liska R., Stampfl J. (2017). Visible Light Photoinitiator for 3D-Printing of Tough Methacrylate Resins. Materials.

[28] Kardar P., Ebrahimi M., Bastani S. (2014). Influence of temperature and light intensity on the photocuring process and kinetics parameters of a pigmented UV curable system. J. Therm. Anal..

[29] Sideridou I., Tserki V., Papanastasiou G. (2002). Effect of chemical structure on degree of conversion in light-cured dimethacrylate-based dental resins. Biomaterials.

[30] Kousaalya A.B., Ayalew B., Pilla S. (2019). Photopolymerization of Acrylated Epoxidized Soybean Oil: A Photocalorimetry-Based Kinetic Study. ACS Omega.

[31] Rusu M.C., Block C., Van Assche G., Van Mele B. (2012). Influence of temperature and UV intensity on photo-polymerization reaction studied by photo-DSC. J. Therm. Anal..

[32] Macarie L., Ilia G. (2005). The influence of temperature and photoinitiator concentration on photoinitiated polymerization of diacrylate monomer. Open Chem..

[33] Zhou H., Li Q., Lee T.Y., Guymon C.A., Jönsson E.S., Hoyle C.E. (2006). Photopolymerization of Acid Containing Monomers: Real-Time Monitoring of Polymerization Rates. Macromolecules.

